# The Humanitarian and the Hooligan

**Published:** 1900-12-08

**Authors:** 


### The Institutional Workshop.

### THE HUMANITARIAN AND THE HOOLIGAN.

It takes many people to make a world, and the observant resident in London can meet if he will representatives of every type within its boundaries in the course of twelve months. The study of the Hooligan question caused the writer to attend a meeting of the Humanitarian League at St. Martin's Town Hall on Tuesday evening, the 4th inst., when Miss Honnor Morten, member of the School Board, was announced to lecture on the Hooligan. Let us first consider the audience. The hall, having accommodation for 700 or 800 people, contained from 150 to 200, who were scattered about, giving an empty appearance. A large number of ladies were present, and the men were a study. We noticed, for instance, that one gentleman, who from his dress and appearance might have been one of the escaped burglars from Borstal Prison, after listening to Miss Morten in despair apparently because she only dealt with infants and young children in Board Schools, which did not interest him at all, left the meeting. A gentleman who sat on the writer's left throughout the whole proceedings talked aloud, expressing his sentiments on the various views enunciated by the speakers. Whenever the suggestion was made to abrogate all law, empty and close all prisons, and abolish personal chastisement, this gentleman expressed his entire agreement with this arrangement, and he became enthusiastic when Mr. Burrows advocated the elimination of the word "punishment" from the English language. To the question: If you abolished all laws and closed all prisons and gave up punishment of every kind, what would you do with the bands of Hooligans who at present perambulate the streets armed with loaded pistols and knives? my neighbour audibly and forcibly stated "science will provide a remedy, if you just leave it to science."

Being struck with the special character of the audience we looked up the objects of the Humanitarian League, which we find to be: "To advocate the principle that it is iniquitous to inflict avoidable suffering on any sentient being."

The lecturer gave a very interesting account of her experience in the Board Schools of London, from which it would appear that they contained nothing that was admirable, and much that was indefensible and contrary to the interests of the children. She made a strong point of the treatment of little mites of infants of three years of age, who were kept in school and not allowed to sleep as they ought to sleep, who were constantly aroused by the mistress, who was in deadly fear of the unexpected arrival of a manager, a school inspector, or some other person to examine into the progress and knowledge of these rising citizens. She asserted that the blue-books contained evidence of these inspections in a report of an Oxford graduate, who informed the Department that these children of three years of age in the schools he inspected showed a lamentable ignorance of mental arithmetic! Her contention was, that under the present system of education all the children, from three years of age and upwards, were kept in a continual state of irritation, so that they became hopelessly restless, could not sit still or remain quiet after some years at a Board School, which was the underlying cause of Hooliganism, and of much that was evil in th rising population of cities. She condemned the reformatory schools, described several that she had visited as "cold hells," from the deadly dulness of everything within their walls, and the absence of everything to make life endurable or happy. Small children were sent to these schools and remained in them for upwards of ten years, to the lasting disgrace of every thinking person in the community, and especially those responsible for education and the children. It was true that one or two small reformatory schools under the School Board were delightful places, but in the whole there was only accommodation for 900 children, and the bright spots were so terribly few as to make the bad ones more appalling and noticeable. Both the lecturer and one of the chief speakers were in favour of the entire abolition of reformatory schools, of the substitution of houses of mercy under kindly, intelligent and humane people, who would bear with the eccentricities of childhood, encourage individual character, and make life restful, helpful and happy. Miss Morten proposed no remedy, but said she would forgive the children for their offences, mistakes and crimes up to seventy times seven. She was apparently in favour of the abolition—as were all the speakers, with one exception—of prisons, of punishment, of the existing laws, and of everything at present done by society to protect itself against individuals and elements, which, if left to themselves, might make life for the majority impossible. All the speakers took credit to themselves that they had been Hooligans when young, and Miss Morten, alluding to the conviction of a child for theft from its mother, said that she and her brothers and sisters used to steal their mother's money, only they did not call it stealing, but a much more reasonable word—which we failed to catch. Finally, she was in favour of moving all the Board Schools out into the country, where the children were apparently to be maintained as boarders at the expense of the ratepayers, and from whence they could return to the cities to their parents for brief holidays once or twice a year. In brief, the lecture consisted of cameo pictures and stories to prove that the School Board system was expensive, mostly wrong, and calculated to create and not prevent Hooliganism, by producing mainly restless, intractable children.

Mr. Burrows, the first speaker, moved a resolution in which it was laid down that one remedy was to enforce the existing laws. He dissented from much that the lecturer had enunciated, and expressed the belief that she would object to the inclusion of a proposal to enforce the existing laws. This proved to be correct, for the Chairman announced that those words would be struck out as being foreign to the spirit of the lecture and the purpose of the meeting, which was accordingly done. Mr. Burrows stuck up for the Board Schools, stated that in the East of London at any rate, where he was a constant visitor, the Board Schools were well conducted, had excellent playgrounds for boys, for girls, and for infants, where anyone could see the children happy and healthily employed. Mr. Burrows asked Miss Morton what she would do with the hundreds of Hooligans with knives and pistols, but got no reply. With reference to the proposal to forgive up to seventy times seven, he was one of those who held that we should by all means forgive everybody everything. He was all for forgiveness; but, having forgiven everybody, would

Miss Morton say how she would handle the armed Hooligans and the criminals? His proposal was to separate the Hooligans from the rest of the community, to place them under suitable conditions to learn a trade and to become useful members of Society.

Mr. Bernard Shaw, the next speaker, expressed himself as against all punitive measures of every kind. He wanted to get rid of the idea of sacrifice from the minds of the people of all nations, whether it was by death in punishment for murder, or the sacrifice of a scapegoat in the shape of a ram or a bullock or a man crucified on wood. Formerly people were content to suffer misery and want in the hope of better times after death, but these beliefs were not held now by the majority of the people. In his view our whole social system was wrong, and we must let matters go on as they were until the Hooligan and the criminal became so threatening and dangerous that Society must deal with them or perish. That meant that Society must so arrange matters that it ceased to breed Hooligans and only bred law-abiding, decent citizens. Mr. Shaw was also against prisons and punishment, especially for the young. He informed the audience that as a youth he was a Hooligan, and that his gang broke into empty houses, though neither he nor his pals stole the furniture nor lifted the valuables. They merely forced the window, and then, taking off their clothes, they rushed about the house in a state of nature until they were tired, when they dressed themselves again, and retired to the bosom of their families, leaving the window open. This interesting adventure, we understand, took place in Ireland. The next speaker professed not to know what had taken the place of the Vestries. He made the only practical suggestion, which was that the new municipalities throughout the Metropolis should at once establish large playgrounds, adequate gymnasiums, and good swimming baths, wherein the rising generation might expend their superfluous energies without damage or annoyance to the older citizens. All the speakers seemed to think that the proposal to found boys' clubs was all very well, but it did not go deep enough. As one speaker persisted in repeating, it was merely a plan to scratch the Hooligan, and, if our observation at the meeting and what we heard there did not deceive us, it needs scratching for many reasons. Altogether we spent a most remarkable and interesting hour at St. Martin's Town Hall.

Mr. Shaw added that the editors of the daily papers in London were mainly responsible for Hooliganism so far as it had excited the apprehension of the people of London. A friend of his who had to be much in the Police Courts took careful statistics of all the cases of crime brought before the Magistrates. He found that the numbers of so-called Hooligan cases, which the newspapers stated had greatly increased, had not really increased at all. What had happened was that the editors of the newspapers had instructed the reporters to give particulars of 5 per cent. more cases of that class than usual. If the editors chose, they could increase every class of crime in the same way, by giving similar instructions to the reporters; for the number of cases of all kinds of crime dealt with was infinitely greater than was ever reported. The public should remember that newspapers were conducted as commercial enterprises, and that the editors had to serve up the kind of news which was fashionable at the moment. Hence the Hooligan being to the fore; certain types of newspapers were full of Hooliganism, because it paid them to publish these particulars. Street ruffianism was not Hooliganism, and it was contrary to the interests of the people for the editors to call all these cases at the present time Hooliganism because the public had this word in its mind.

